# Metabolic Impairment in Coronary Artery Disease: Elevated Serum Acylcarnitines Under the Spotlights

**DOI:** 10.3389/fcvm.2021.792350

**Published:** 2021-12-16

**Authors:** Joséphine Gander, Justin Carrard, Hector Gallart-Ayala, Rébecca Borreggine, Tony Teav, Denis Infanger, Flora Colledge, Lukas Streese, Jonathan Wagner, Christopher Klenk, Gilles Nève, Raphael Knaier, Henner Hanssen, Arno Schmidt-Trucksäss, Julijana Ivanisevic

**Affiliations:** ^1^Division of Sports and Exercise Medicine, Department of Sport, Exercise and Health, University of Basel, Basel, Switzerland; ^2^Metabolomics Platform, Faculty of Biology and Medicine, University of Lausanne, Lausanne, Switzerland; ^3^Division of Sports Science, Department of Sport, Exercise and Health, University of Basel, Basel, Switzerland

**Keywords:** metabolomics, coronary artery disease, carnitine, acylcarnitine, branched-chain amino acids, fatty acid oxidation (FAO), mitochondria

## Abstract

Coronary artery disease (CAD) remains the leading cause of death worldwide. Expanding patients' metabolic phenotyping beyond clinical chemistry investigations could lead to earlier recognition of disease onset and better prevention strategies. Additionally, metabolic phenotyping, at the molecular species level, contributes to unravel the roles of metabolites in disease development. In this cross-sectional study, we investigated clinically healthy individuals (*n* = 116, 65% male, 70.8 ± 8.7 years) and patients with CAD (*n* = 54, 91% male, 67.0 ± 11.5 years) of the COmPLETE study. We applied a high-coverage quantitative liquid chromatography-mass spectrometry approach to acquire a comprehensive profile of serum acylcarnitines, free carnitine and branched-chain amino acids (BCAAs), as markers of mitochondrial health and energy homeostasis. Multivariable linear regression analyses, adjusted for confounders, were conducted to assess associations between metabolites and CAD phenotype. In total, 20 short-, medium- and long-chain acylcarnitine species, along with L-carnitine, valine and isoleucine were found to be significantly (adjusted *p* ≤ 0.05) and positively associated with CAD. For 17 acylcarnitine species, associations became stronger as the number of affected coronary arteries increased. This implies that circulating acylcarnitine levels reflect CAD severity and might play a role in future patients' stratification strategies. Altogether, CAD is characterized by elevated serum acylcarnitine and BCAA levels, which indicates mitochondrial imbalance between fatty acid and glucose oxidation.

## Introduction

Coronary artery disease (CAD) remains the leading cause of death worldwide (1). In spite of this, in clinical practice, patients' biochemical stratification is still mainly limited to total cholesterol and triglyceride quantification (2). Following recent advances in mass spectrometry and bioinformatics, high-throughput and high-coverage metabolomic approaches now provide more precise metabolic profiling at the molecular species level (3). Upgrading patients' metabolic phenotyping could lead to earlier recognition of disease onset, optimization of prevention strategies and health monitoring, ultimately reducing CAD-related burden (4, 5). In addition to biomarker discovery, patients' metabolic phenotyping is of utmost importance to decipher the roles of metabolites in disease development (6).

Metabolites, including acylcarnitines and amino acids, have long been used to diagnose inborn errors of metabolism (7, 8). Recently, acylcarnitines have been suggested as potential biomarkers of cardiometabolic diseases (9). Specifically, elevated acylcarnitines levels were observed in patients with type 2 diabetes (10, 11), heart failure (12–14), and in middle-aged adults and elderly with a combination of different cardiovascular diseases (15, 16). Additionally, circulating acylcarnitines were associated with the risk of myocardial infarction and cardiovascular death in individuals with stable angina pectoris (17) as well as with the risk of cardiovascular events at 3-year follow-up in patients with CAD (18, 19). Similarly, elevated serum concentration of branched-chain amino acids (BCAAs), whose metabolism is tightly related to that of short-chain acylcarnitines, was observed in patients with insulin resistance (20–22), obesity (23, 24), diabetes (25–27), dyslipidemia, and CAD (19, 28, 29). These studies, however, investigated patients already suffering from cardiometabolic diseases without including healthy controls (12, 13, 15, 17–19, 23–28) or with poorly characterized controls (14, 16, 20, 22, 29). Comparing diseased against healthy metabolic signature is essential to reveal disease-associated alterations and to improve our understanding of metabolites' roles in pathophysiological processes (30, 31). Furthermore, these studies investigating a limited number of acylcarnitines with a maximum of 12 species detected (19). This number can now be outranged due to technological advancements (32).

Acylcarnitines play a key role in the transport of fatty acids longer than 10 carbon atoms (C) across mitochondrial membranes for oxidation (**Figure 5**) (37, 46). Reduced oxygen availability impairs fatty acid oxidation, with consequent accumulation of acyl-CoA and acylcarnitines in mitochondria (40, 47, 48). Accumulation of long-chain acylcarnitines further disrupts membrane function and energy metabolism, which ultimately leads to cellular stress, inflammation, insulin resistance and cardiac arrhythmias (32, 44, 45). In contrast to medium- and long-chain acylcarnitines (C6–C22), whose accumulation is mainly related to impaired fatty acid oxidation, short- and odd-chained acylcarnitines (C3 and C5) usually derive from disrupted BCAA catabolism (**Figure 5**) (43–45). Thus, acylcarnitines and BCAAs, which appear to be promising indicators of mitochondrial function and cardiometabolic health, could be used to improve patients' metabolic phenotyping.

This cross-sectional population-based study quantified a large panel of circulating short-, medium- and long-chain acylcarnitines as well as BCAAs within well-characterized clinically healthy individuals and patients with CAD of the COmPLETE study (49). Our approach applied a high-coverage targeted hydrophilic interaction liquid chromatography coupled with high resolution mass spectrometry (HILIC-HRMS) (32). The acquired comprehensive metabolic profiles allowed us to identify a set of acylcarnitines and BCAAs associated with CAD.

## Materials and Methods

### Study Design and Participants

Subsets of the COmPLETE-Health (*n* = 116, mean age 70.8 ± 8.7 years old, 65% male) and COmPLETE-Heart samples (*n* = 54, mean age 67.0 ± 11.5 years old, 91% male) were investigated. As reported in the study protocol, only clinically healthy participants from the Basel area (Switzerland), who had no exercise-limiting chronic diseases and were non-smokers (or had quit at least 10 years previously) were included in the COmPLETE-Health sample. This excluded participants with a history of CAD, stroke, heart failure, lower-extremity artery disease, any kind of malignant tumor, diabetes, obesity, clinically apparent kidney failure, severe liver disease, chronic obstructive pulmonary disease GOLD stages two to four, arterial hypertension grades two and three, drug or alcohol abuse, exercise-limiting osteoporosis or musculoskeletal conditions and clinically manifest Alzheimer's disease or dementia. The investigated subset of the COmPLETE-Heart sample consisted exclusively of patients suffering from CAD, diagnosis of which was confirmed by senior cardiologists. Patients with unstable angina pectoris, uncontrolled brady- or tachyarrythmia, permanent atrial fibrillation, severe valvular disease, acute myocardial infarction, transient ischemic attack, or stroke in the last 3 months were excluded. The exact recruitment procedure and the full list of inclusion and exclusion criteria can be found in the COmPLETE study protocol (49). The COmPLETE study was funded by the Swiss National Science Foundation (Grant No. 182815) and approved by the Ethics Committee of North-Western and Central Switzerland (EKNZ 2017-01451). A written informed consent document was obtained from all participants prior to inclusion.

### Data Sources

Data collection was carried out between January 2018 and June 2019. Medical history and medication were reviewed by a physician using a standardized questionnaire. Participants were randomly allocated to one of five time slots (08:00, 10:00, 12:00, 14:00 and 16:00) for the measurements, which took around 4 h in total. They were instructed not to diverge from habitual eating behavior (for the previous 72 h), to avoid exercising, drinking alcohol (for the previous 24 h) and drinking caffeinated beverages (for the previous 4 h). On the day of sample collection, participants took the prescribed medication as usual. Fasting blood samples (at least 3 h fasting time) were collected before any kind of measurements involving physical activity. Trained medical staff collected serum samples (2 × 7.5 mL serum-gel, Monovette®, Sarstedt, Nümbrecht, Germany) by venipuncture in the cubital fossa. Serum samples were slightly shaken for 30 min, centrifuged (3,000 rpm; 10 min; 20–23°C) and aliquoted before being frozen at −80°C.

### Anthropometric Values

Height and body mass were measured to the nearest 0.5 cm and 0.1 kg, respectively. Body mass index was calculated as kg/m^2^. Body composition was analyzed using a four-segment bioelectrical impedance analysis (Inbody 720, Inbody Co. Ltd., Seoul, South Korea). Using InBody 720, -as opposed to dual-energy x-ray absorptiometry analysis, to measure appendicular muscle mass was judged to be acceptable (50). After a rest phase of 10 min, resting systolic and diastolic blood pressures and resting heart rate were measured in supine position using a non-invasive vascular screening system (VaSera VS-1500 N; Fukuda Denshi, Tokyo, Japan). The peak oxygen uptake (VO_2_ peak) was used as marker for the cardiorespiratory fitness and was determined during an exercise test until maximal exertion (i.e., volitional exertion, dyspnea, or fatigue) using an electromagnetically braked cycle ergometer (Ergoselect 200; Ergoline, Bitz, Germany) and a computer-based system (MetaMax 3B; Cortex Biophysik GmbH, Leipzig, Germany). VO_2_ peak was defined as the highest 30 s average of VO_2_ at any point of the test.

### Biochemical Analysis

Serum samples were analyzed for triglyceride, total cholesterol, high-density lipoprotein (HDL) and low-density lipoprotein (LDL) cholesterol concentrations using an Olympus AU680 automatic analyzer (Beckman Coulter, Brea, CA, USA), enzymatic reagents (DiaSys, Holzheim, Germany) and secondary standards (Roche Diagnostics, Mannheim, Germany). For glycated hemoglobin (HbA1c), whole blood was analyzed by high pressure liquid chromatography (HPLC) using D-10 (Bio-Rad, Hercules, CA, USA). NT-proBNP was determined using a chemiluminescent microparticle immunoassay (Architect, Abott, IL, United States).

### Metabolic Profiling

A large panel of carnitine related metabolites, including free carnitine, deoxycarnitine and 36 acylcarnitine species (Supplementary Table 1) were targeted in serum samples. BCAAs were also measured, as C3- and C5-acylcarnitine species are byproducts of the BCAA catabolism (43–45). Analysis was conducted at the Metabolomics Platform, Faculty of Biology and Medicine, University of Lausanne (Switzerland). A detailed description of the method used is available (32).

#### Sample Preparation

For absolute quantification of acylcarnitines and BCAAs, samples were prepared by mixing 20 μL of serum with 250 μL of ice-cold methanol spiked with internal standard (IS) solution of corresponding isotopically labeled acylcarnitines and BCAAs (Supplementary Table 1), which was completed to 300 μL with 0.1% formic acid in water. Samples were then mixed by shaking and centrifuged for 15 min at 4°C and 2,700 g. The resulting supernatants were transferred to LC-MS vials prior to injection.

#### Metabolite Quantification

Extracted samples were analyzed by HILIC-HRMS in full scan MS mode using a Q Exactive™ Hybrid Quadrupole-Orbitrap interfaced with the ultra-high-performance liquid chromatography (UHPLC) Vanquish Horizon (Thermo Fisher Scientific) as previously described (32). Metabolites were separated using an ethylene bridged hybrid (BEH) amide column (1.7 μm, 100 mm × 2.1 mm I.D.) (Waters, MA, US) in positive ionization mode. The mobile phase was composed of A = 20 mM ammonium formate and 0.1% formic acid (FA) in water and B = 0.1% FA in acetonitrile (ACN). The gradient elution started at 95% B (0–2 min) decreasing to 65% B (2–14 min), reaching 50% B at 16 min and was followed by an isocratic step (16–18 min) before a 4 min post-run for column re-equilibration. The flow rate was 400 μL/min, column compartment 25°C and the sample injection volume was 2 μl. Heated electrospray ionization (HESI) source conditions were set as follows; sheath gas flow at 60, aux gas flow rate at 20, sweep gas flow rate at 2, spray voltage at +3 kV, capillary temperature at 300°C, s-lens RF level at 60 and aux gas heater temperature at 300°C. Full scan HRMS data was acquired over the *m/z* range 50–750, with the following MS acquisition parameters; mass resolving power at 70,000 full width at half maximum (FWHM), 1 microscan, 1e6 automatic gain control (AGC) and maximum inject time at 100 ms.

#### Data Processing and Analysis

Raw data files were processed using Xcalibur 4.1 (Thermo Fisher Scientific). Peak was manually curated and corrected if necessary. For absolute quantification, calibration curves and the stable isotope spike (or internal standard spike) at known concentration were used to report the concentrations quantified in each serum sample. Linearity of the standard curves was evaluated for each metabolite using 11-point range. A human plasma standard reference material (Certificate of Analysis, NIST 1950) was analyzed within each batch of samples and used as a quality control for the validation of measurement accuracy.

### Statistical Methods

Metabolite concentrations were log2-transformed and z-standardized prior to statistical analysis. Multiple linear regressions were run to assess associations between metabolites and CAD phenotype. To determine which confounders required adjustment for regressions, directed acyclic graph (DAG) were drawn (Supplementary Figure 1) (51, 52). Metabolites were used as dependent variables, while CAD phenotype and confounders served as independent variables. Regressions using acylcarnitines or carnitine as dependent variable were adjusted for the following confounders age (53, 54), sex (55), HbA1c (%) (11, 56, 57), body fat (%) (11, 58, 59), smoking habits (60), antihypertensive and lipid lowering medication (61–64) as well as fasting and sampling time (Supplementary Figure 1) (65–67). Regressions using BCAAs as dependent variable were adjusted for the following confounders: age, sex (67–70), skeletal muscle mass (71, 72), smoking habits (60, 73), sampling time and fasting time (Supplementary Figure 2) (65–67).

Two sets of multiple linear regressions were run. In the first set, CAD was defined as a categorical two-level variable opposing sickness vs. health. In the second set, CAD was defined as the number of stenosed coronary arteries (0, 1, 2, or 3). As the concentrations of deoxycarnitine, hydroxyhexanoylcarnitine (C6:0-OH), hydroxydodecanoylcarnitine (C12:0-OH) and arachidonylcarnitine (C20:4) were below the quantification limit (0.003 μM) of the HILIC-HRMS method employed for some participants (deoxycarnitine n=1, hydroxyhexanoylcarnitine *n* = 2, hydroxydodecanoylcarnitine *n* = 1, arachidonylcarnitine *n* = 1), a Tobit regression using the CensReg R package was applied for these metabolites to estimate regression coefficients in the presence of left censored values (74).

Graphical methods were used to assess linearity, normal distribution, and homoscedasticity of data. *P*-values were adjusted using the Benjamini-Hochberg (BH) method, separately within each set of multiple linear regression (75). Adjusted *p* ≤ 0.05 were considered as significant. Statistical analyses were carried out using R (version 4.0.2) (76). Rain plots were computed using a previously published R-code (77).

## Results

### Participants' Characteristics

The investigated subjects consisted of 116 healthy individuals (70.8 ± 8.7 years, 65% male) and 54 patients with confirmed CAD (67 ± 11.5 years, 91% male) (Table 1). The clinically healthy individuals had normal body mass index (BMI) and HbA1c values. (78). Mean blood pressure values were in the high normal range, with 20% of participants treated for arterial hypertension grade one (79). Sixty-six percent of the clinically healthy individuals had never smoked, whereas 34% had quit smoking more than 10 years previously. Both clinically healthy controls and CAD patients were characterized by elevated LDL-cholesterol according to the 2019 ESC/EAS guidelines on primary (clinically healthy participants) and secondary (CAD patients) prevention (2). Eighty nine percentage of the CAD patients were under statin, compared to only 8% of the clinically healthy participants, which could explain the lower levels of LDL-cholesterol in the CAD patients. CAD patients displayed elevated NT-proBNP levels and a mean BMI value in the overweight range, while HbA1c, triglyceride and HDL-cholesterol levels as well as systolic and diastolic blood pressure values were normal (2, 78–80). It is worth noting that all CAD patients were on antihypertensive medications. Additionally, 50% of CAD patients were non-smokers, while 20% ceased smoking at least 10 years ago. Fasting duration was of at least 3 h with mean of 8.5 ± 5.3 h for those with CAD and 6.7 ± 3.0 h for clinically healthy individuals.

**Table 1:** Participants' characteristics.

	**Clinically healthy**	**CAD**	***P*-value (*t*-test or Mann Whitney *U* test)**	***P*-value (chi-squared test**
**Participants**, ***n*** **(%)**	116 (68)	54 (32)		
**Anthropometry, mean** **±** **SD**				
Age (years)	70.8 ± 8.7	67.0 ± 11.5	0.034	
Male (%)	64.7%	90.7%		<0.001
Body mass (kg)	67.0 ± 10.4	84.0 ± 14.9	<0.001	
Body fat (%)	27.9 ± 6.9	30.2 ± 6.8	0.043	
Lean mass (kg)	26.3 ± 5.1	32.2 ± 5.5	<0.001	
BMI (kg/m^2^)	24.0 ± 2.8	27.8 ± 4.1	<0.001	
Systolic blood pressure (mmHg)	131.8 ± 13.2	126.7 ± 15.4	0.029	
Diastolic blood pressure (mmHg)	80.8 ± 8.3	77.3 ± 10.8	0.024	
VO_2_peak (L/min)	1.86 ± 0.53	1.82 ± 0.60	0.629	
**Smoking status**, ***n*** **(%)**				
Never smoked	76 (66)	27 (50)		0.019
Smokers	0 (0)	6 (11)		<0.001
Ex-smokers (quit <10 years ago)	40 (34)	11 (20)		<0.001
Ex-smokers (quit >10 years ago)	0 (0)	10 (19)		0.110
**Biochemical parameters, mean** **±** **SD**				
Fasting duration prior to blood sampling (h)	6.7 ± 3.0	8.5 ± 5.3	0.522	
Total cholesterol (mmol/L)	6.17 ± 1.04	4.12 ± 0.82	<0.001	
LDL-C (mmol/L)	3.46 ± 0.68	2.16 ± 0.52	<0.001	
HDL-C (mmol/L)	3.47 ± 0.68	2.16 ± 0.52	<0.001	
Triglycerides (mmol/L)	1.37 ± 0.76	1.50 ± 0.96	0.611	
HbA1c (%)	5.4 ± 0.3	6.1 ± 0.7	<0.001	
NT-ProBNP (pg/ml)	145.5 ± 110.4	603.0 ± 651.4	<0.001	
**Comorbidities**				
Hypertension	23 (20)	54 (100)		<0.001
**No of coronary artery with stenosis**, ***n*** **(%)**				
0	116 (100)	0 (0)		
1	0 (0)	12 (22)		
2	0 (0)	15 (28)		
3	0 (0)	24 (44)		
Not known	0 (0)	3 (6)		
Diabetes mellitus	0 (0)	10 (19)		<0.001
**Cardiovascular medications**, ***n*** **(%)**				
Antihypertensive	23 (20)	54 (100)		<0.001
ACE inhibitors	3 (3)	32 (59)		<0.001
Angiotensin receptor blockers (ARBs)	19 (16)	18 (33)		<0.001
Amlodipin	6 (5)	7 (13)		0.008
Beta-blockers	4 (3)	43 (80)		<0.001
Statins	9 (8)	48 (89)		<0.001
**Diabetes medications**, ***n*** **(%)**				
Oral antidiabetic drugs	0 (0)	9 (17)		<0.001
Insulin	0 (0)	6 (11)		<0.001
**Other medications**, ***n*** **(%)**	52 (45)	52 (96)		

*A Student's t-test was performed to compare body fat, lean mass, BMI, systolic and diastolic blood pressure between clinically healthy and sick individuals. Other continuous variables were compared using a Mann-Whitney U test. A chi-squared. BMI, body mass index; LDL-C, low density lipoprotein cholesterol; HDL-C, low density lipoprotein cholesterol; HbA1c, glycated hemoglobin; NT-proBNT, N-terminal (NT)-pro hormone B-type natriuretic peptide. Other drugs include: Acetylsalicylic acid (57), diuretics (36), anticoagulants/antiplatelets (32), vitamins (32), proton-pump inhibitors (30), chondroitinsulfat (13), lipid-lowering drugs except statins (11), non-steroidal anti-inflammatory drugs (11), thyroid hormones (11), topical ophthalmic drugs (11), estrogen/hormone replacement therapy (9), 5α-reductase inhibitors (7), paracetamol (5), uricostatic drugs (5), antidepressants (3), antihistamines (3), bisphosphonate (3), ginkgo (3), non-benzodiazepine benzodiazepine receptor agonists (3), fluticasone/salmeterol (2), prednisolone (2), pregabalin (2), benzodiazepine (1), febuxostatum (1), fluticasone/vilanterol (1), gabapentin (1), L-dopa/benserazid (1), melatonin (1), mesalazine (1), molsidomin (1), mometasone (1), polystyrene sulfonate (1), tamsulosin (1), topic fluticasone (1), tiotropium (1), rifamycin (1)*.

### Associations Between Circulating Acylcarnitines and CAD

**Figure 1:**
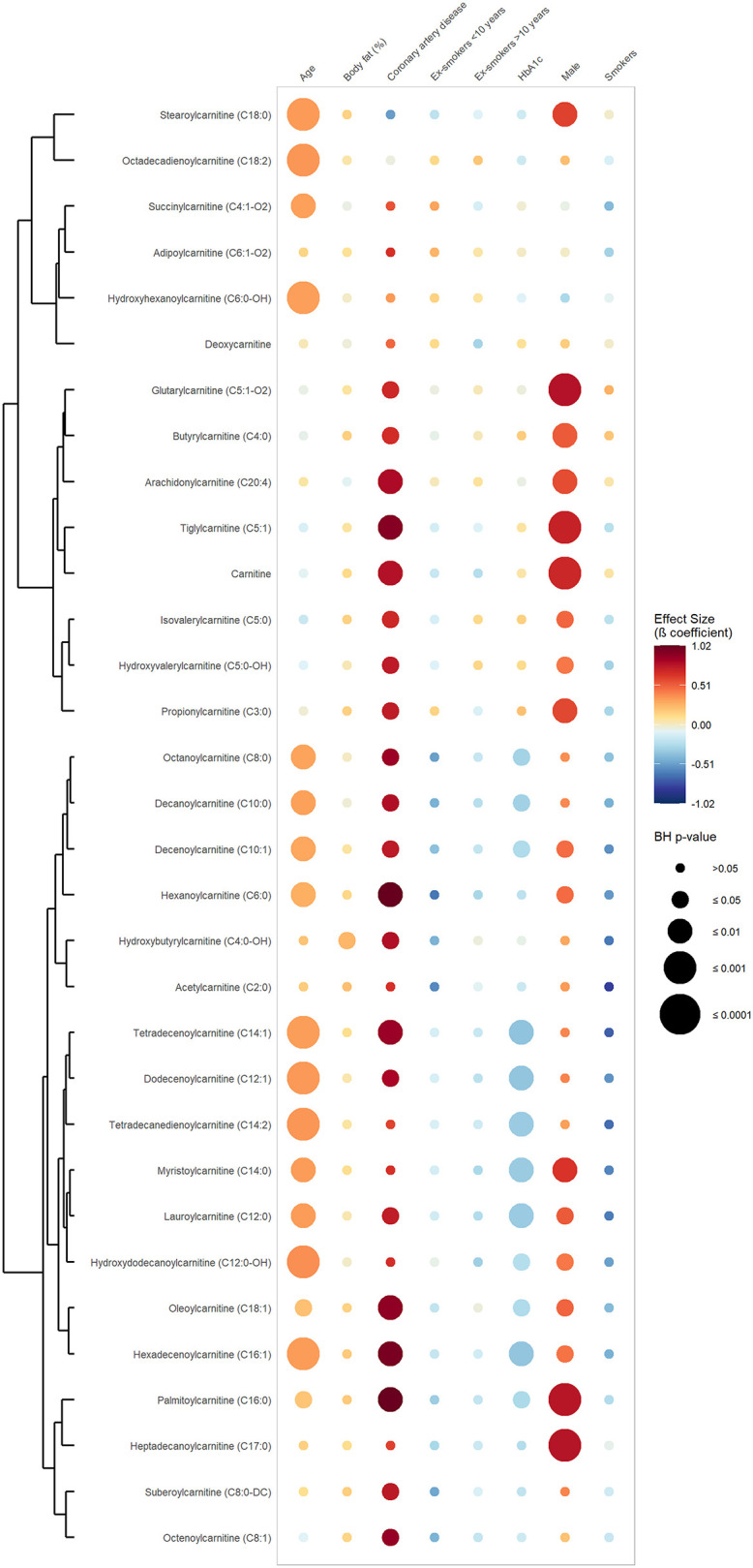
Associations between serum carnitine/acylcarnitine species, coronary artery disease and selected cardiovascular risk factors. This rainplot represents the results of the first set of regression, in which metabolites were used as dependent variables (vertical axis), while CAD phenotype (two-level variable opposing sickness vs. health) and confounders served as independent variables (horizontal axis). The redder the dots the higher the beta coefficient and the bigger the dot the smaller the adjusted *p*-value. A clustering has been done regrouping the metabolites with similar beta-coefficients and adjusted *p*-values. BH, Benjamini-Hochberg; HbA1c, glycated hemoglobin.

**Figure 2:**
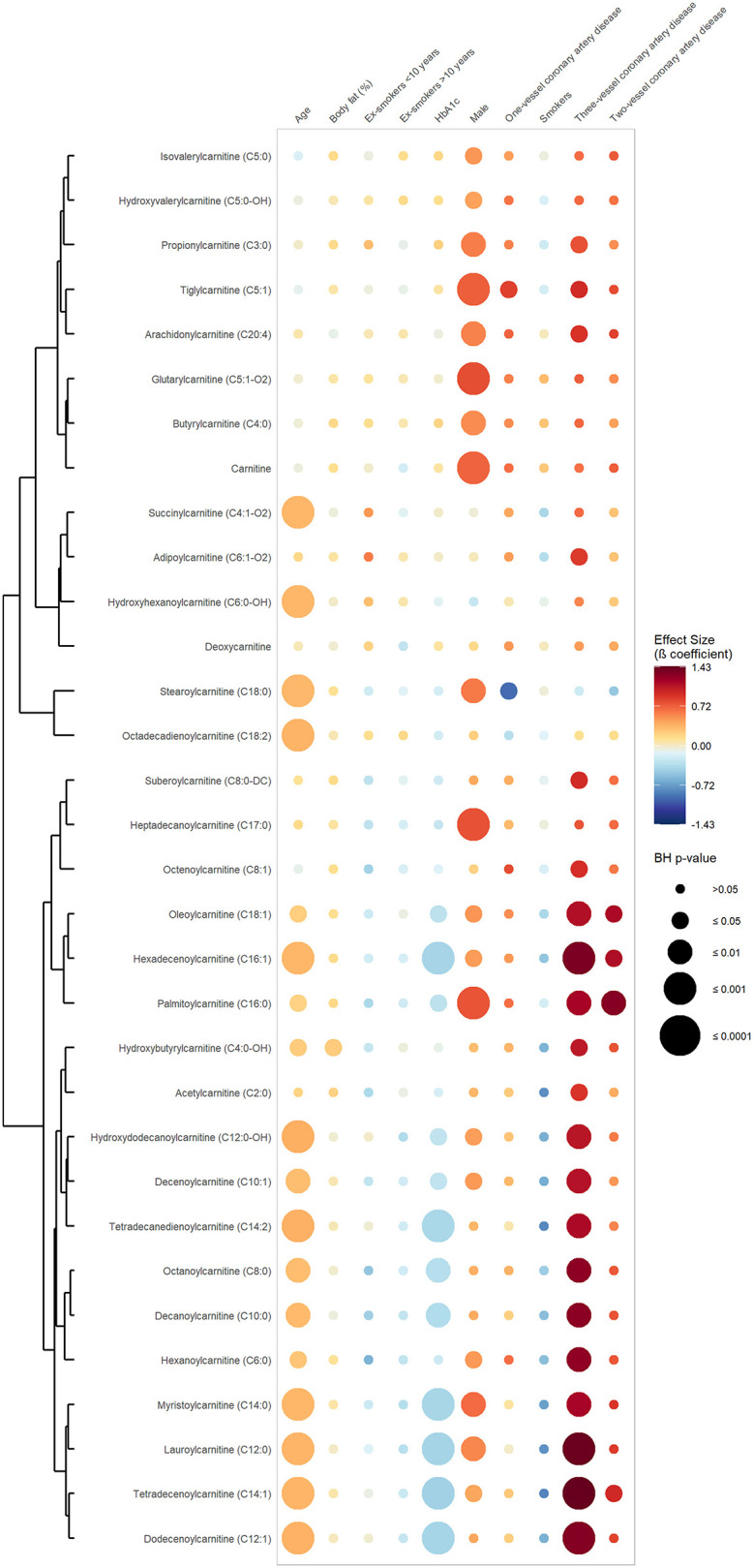
Associations between serum carnitine/acylcarnitine species, number of stenosed coronary arteries and selected cardiovascular risk factors. This rainplot represents the results of the second set of regression, in which metabolites were used as dependent variables (vertical axis), while the number of stenosed coronary arteries (0, 1, 2, or 3) and confounders served as independent variables (horizontal axis). The redder the dots the higher the beta coefficient and the bigger the dot the smaller the adjusted *p*-value. A clustering has been done regrouping the metabolites with similar beta-coefficients and adjusted *p*-values. BH, Benjamini-Hochberg; HbA1c, glycated hemoglobin.

Figure 1 exhibits the results of the first set of regression, in which CAD was defined as a dichotomous variable opposing CAD vs. absence of CAD. It shows that 20 out of 30 quantified carnitines (seven short-chain, eight medium-chain and five long-chain acylcarnitines) were significantly and positively associated with CAD phenotype after adjustment for confounders (Table 2, Supplementary Table 2, Supplementary Figure 3). Hexanoylcarnitine (C6:0) showed the strongest positive association with CAD (β-coefficient 1.02; BH *p* ≤ 0.004) followed by palmitoylcarnitine (C16:0) (β-coefficient 1.02; BH *p*-value 0.002), and hexadecenoylcarnitine (C16:1) (β-coefficient 0.96; BH *p*-value 0.003) (Supplementary Table 2). Figure 2 represents the results of the second set of regressions, in which CAD was defined as the number of stenosed coronary arteries (zero-, one-, two- and three-vessel coronary artery disease). It shows that the strength of association (β-coefficient) increased with increasing number of affected coronary arteries for 17 acylcarnitines (3 short-, 8 medium- and 6 long-chain acylcarnitine species) (Supplementary Table 2). Besides, individuals with CAD had significant elevated levels of two short-chain hydroxylated acylcarnitines (C4:0-OH and C5:0-OH) and dicarboxylic acylcarnitine suberoylcarnitine (C8:0-DC) (Supplementary Table 2).

**Table 2:** Quantified metabolites.

	**Abbreviation**	**Targeted metabolites**
	C0	Carnitine
		Deoxycarnitine
Short-chain (*n* = 9)	C2:0	Acetylcarnitine
	C3:0	Propionylcarnitine
	C4:0	Butyrylcarnitine
	C4:0-OH	Hydroxybutyrylcarnitine
	C4:1-O2	O-succinylcarnitine
	C5:0	Isovalerylcarnitine
	C5:0-OH	3-Hydroxyvalerylcarnitine
	C5:1	Tiglylcarnitine
	C5:1-O2	Glutarylcarnitine
Medium-chain (*n* = 11)	C6:0	Hexanoylcarnitine
	C6:0-OH	3-Hydroxyhexanoylcarnitine
	C6:1-O2	Adipoylcarnitine
	C8:0	Octanoylcarnitine
	C8:0-DC	Suberoylcarnitine
	C8:1	2-Octenoylcarnitine
	C10:0	Decanoylcarnitine
	C10:1	Trans-2-decenoylcarnitine
	C12:0	Lauroylcarnitine (dodecanoylcarnitine)
	C12:0-OH	3-Hydroxydodecanoylcarnitine
	C12:1	Trans-2-dodecenoylcarnitne
Long-chain (*n* = 10)	C14:0	Myristoylcarnitne (tetradecanoylcarnitine)
	C14:1	Trans-2-tetradecenoylcarnitine
	C14:2	Cis, cis-5,8-tetradecanedienoylcarnitine
	C16:0	Palmitoylcarnitine (hexadecanoylcarnitine)
	C16:1	Trans-2-hexadecenoylcarnitine
	C17:0	Heptadecanoylcarnitine
	C18:0	Stearoylcarnitine (octadecanoylcarnitine)
	C18:1	Oleoylcarnitine (octadecenoylcarnitine)
	C18:2	Cis, cis-9,12- octadecadienoylcarnitine
	C20:4	Arachidonylcarnitine
BCAA		Leucine
		Isoleucine
		Valine

*Acylcarnitines can be categorized depending on the number of carbon atoms of their acyl-group into short-chain (C2–C5), medium-chain (C6–C13), and long-chain (C14–C21) acylcarnitines (34, 80). C, number of carbon atoms of the acyl-group; DC, dicarboxyl; OH, Hydroxy; BCAA, branched-chain amino acid*.

Regarding the cardiometabolic risk factors used as confounders, age showed significant and positive associations with more than 50% of the measured acylcarnitine species (one short-, eight medium- and eight long-chain species, including two hydroxylated acylcarnitines). Six medium-chain and six long-chain acylcarnitines were found to be significantly and negatively associated with HbA1c, while no significant association was found between acylcarnitines and smoking status.

### The BCAA Signature of CAD

**Figure 3:**
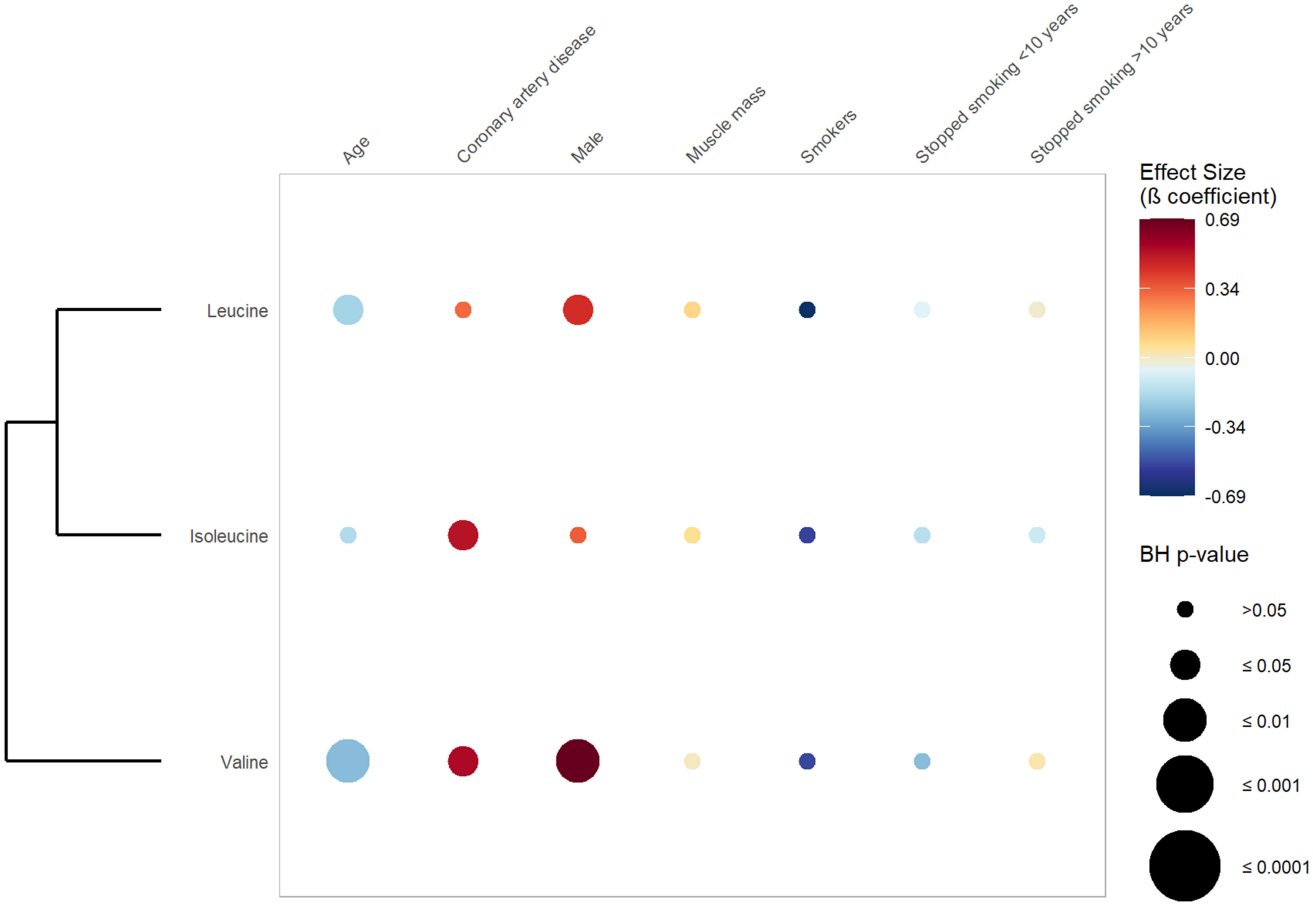
Associations between serum branched-chain amino acids, coronary artery disease and confounders. This rainplot represents the results of the first set of regression, in which metabolites were used as dependent variables (vertical axis), while CAD phenotype (two-level variable opposing sickness vs. health) and confounders served as independent variables (horizontal axis). The redder the dots the higher the beta coefficient and the bigger the dot the smaller the adjusted *p*-value. A clustering has been done regrouping the metabolites with similar beta-coefficients and adjusted *p*-values. BH, Benjamini-Hochberg; HbA1c, glycated hemoglobin.

**Figure 4:**
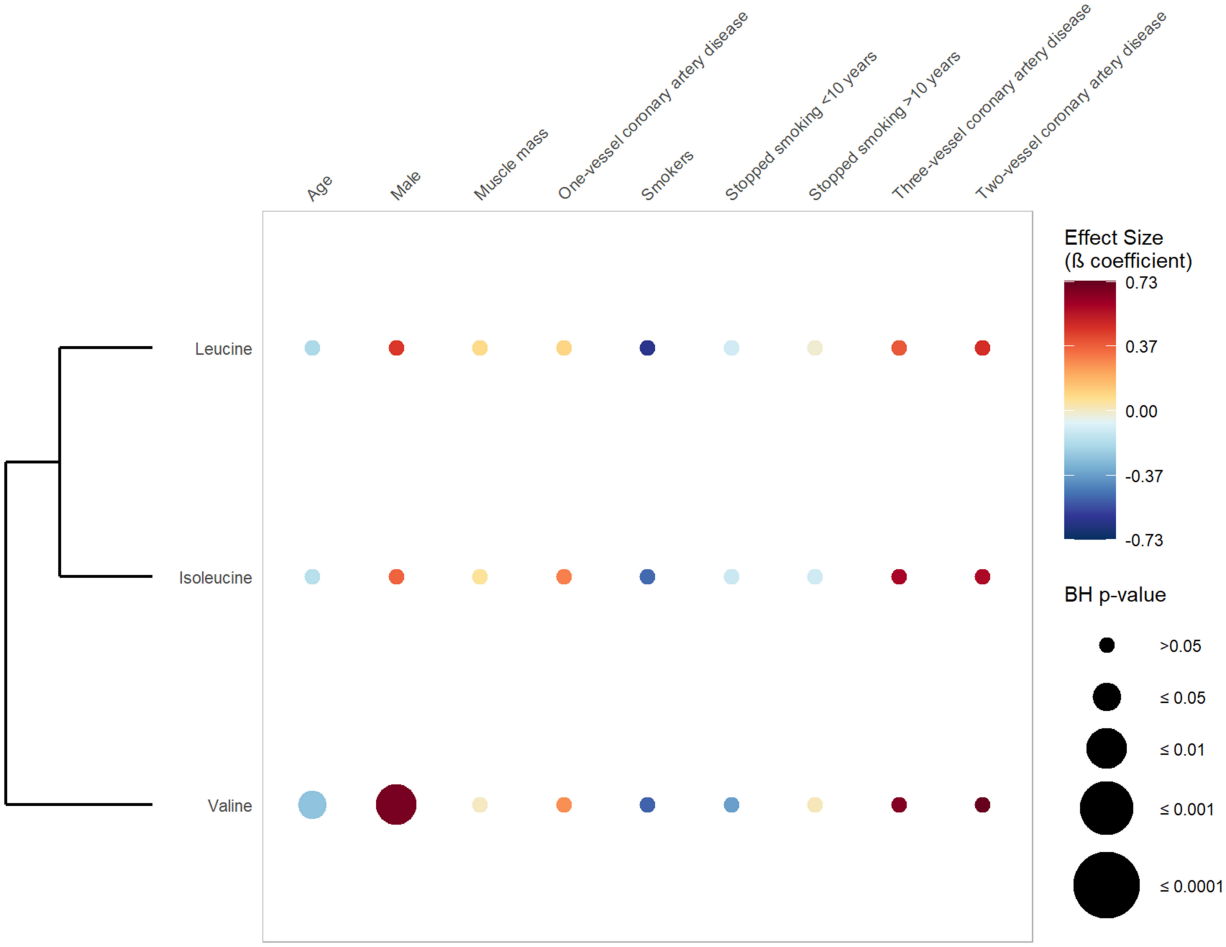
Association between serum branched-chain amino acids, number of stenosed coronary arteries and confounders. This rainplot represents the results of the second set of regression, in which metabolites were used as dependent variables (vertical axis), while the number of stenosed coronary arteries (0, 1, 2, or 3) and confounders served as independent variables (horizontal axis). The redder the dots the higher the beta coefficient and the bigger the dot the smaller the adjusted *p*-value. A clustering has been done regrouping the metabolites with similar beta-coefficients and adjusted *p*-values. BH, Benjamini-Hochberg; HbA1c, glycated hemoglobin.

Figure 3 exhibits the results of the first set of regression, in which CAD was defined as a dichotomous variable opposing CAD vs. absence of CAD. It shows that valine and isoleucine were significantly and positively associated with CAD phenotype (valine: β-coefficient 0.55; BH *p*-value 0.046 and isoleucine: β-coefficient 0.52; BH *p*-value 0.046) (Supplementary Table 2). Conversely, no significant association was found between BCAAs and the number of stenosed coronary arteries (Figure 4). Concerning the cardiometabolic risk factors used as confounders, valine and leucine showed a significant negative association with age and a significant positive association with the male sex. No significant association was found between muscle mass and BCAAs.

## Discussion

The present study revealed significant elevated levels of circulating acylcarnitines and BCAAs in patients with CAD compared to clinically healthy individuals. Acylcarnitine species of all chain-length showed positive associations with CAD phenotype. Compared to previous studies, the present work quantified a larger panel of acylcarnitines, which allowed the identification of novel associations between acylcarnitine species and CAD phenotype (17–19). For instance, the short- and medium-chain acylcarnitines C4:0, C4:0-OH, C5:0-OH, C5:1, C5:1-O2, C6:0, and C8:1 were observed for the first time to be associated with CAD. To the authors' best knowledge, this is the first study to have examined associations between the number of stenosed coronary arteries and circulating acylcarnitines and BCAAs. For 17 acylcarnitine species, associations became stronger as the number of affected coronary arteries increased. The number of coronary artery disease has been described as a simple measure of CAD severity (81–84). Globally, the higher the number of affected coronary arteries, the higher the probability that a bigger part of the myocardium could be damaged (85, 86). Therefore, there is a relation between the number of affected coronary arteries, the area of impaired myocardium and heart function. This implicates that circulating acylcarnitine levels might reflect CAD severity.

### Elevated Medium- and Long-Chain Acylcarnitine Levels in CAD

**Figure 5:**
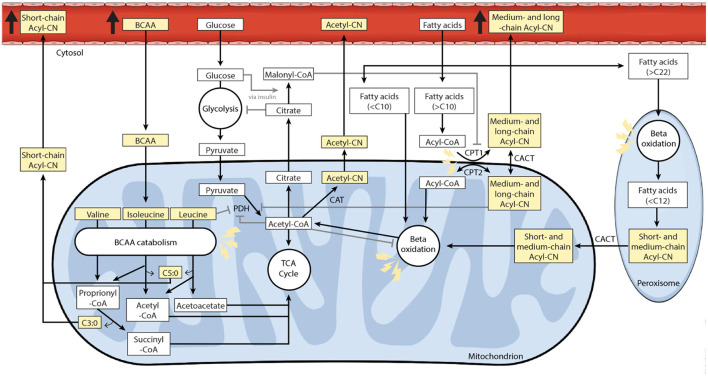
Acylcarnitine and branched-chain amino acid (BCAA) metabolism in a cardiac cell. This study found elevated levels of circulating short-, medium- and long-chain acylcarnitine and BCAA species in patients with CAD compared to clinically healthy individuals. Under aerobic conditions, lipids represent the main energetic substrate in cardiac cells (33). The main role of carnitine and acylcarnitines is to transport fatty acids, containing acyl-chain(s) of 10 or more carbon atoms, into the mitochondria for subsequent beta-oxidation. The so-called carnitine shuttle includes several enzymes. The enzyme carnitine palmitoyltransferase 1 (CPT1) located at the outer mitochondrial membrane converts acyl-CoAs into acylcarnitines. These are then transported through the inner mitochondrial membrane by the carrier carnitine/acylcarnitine translocase (CACT). Once inside the mitochondrion, the enzyme carnitine palmitoyltransferase 2 (CPT2) converts acylcarnitines back to their corresponding acyl-CoAs, which will then undergo beta-oxidation to produce acetyl-CoA (34–36). Beyond fuel trafficking, acylcarnitines also defend against mitochondrial stress by buffering the intracellular free CoA to acyl-CoA ratio (37–39). The carnitine shuttle enables mitochondrial export of excess carbons in the form of acylcarnitines, which can then be excreted via blood and urine (37, 39). This process also supports metabolic flexibility by relieving the inhibition of PDH induced by acetyl-CoA accumulation (40). Metabolic flexibility is the ability to switch between substrate for energy production depending on substrate availability (41). Fatty acids and glucose intermediates compete as metabolic substrate for energy production in cardiac mitochondria (Randle cycle) (42). Short- and odd-chain acylcarnitine species, such as propionylcarnitine (C3) and isovalerylcarnitine (C5), are usually derived from BCAA catabolism (43–45). Molecules on a yellow background were measured in this study. Regulatory mechanisms are represented with gray lines, normal arrows for stimulation and arrows to bar for inhibition. Lightning icons represent impairment. Acyl-CN, acylcarnitine; BCAA, branched-chain amino acid; C, number of carbon atoms; CACT, carnitine-acylcarnitine translocase; CAT, carnitine acetyltransferase; CPT1, carnitine palmitoyltransferase 1; CPT2, carnitine palmitoyltransferase 2; PDH, pyruvate dehydrogenase.

In the present study, circulating medium- and long-chain acylcarnitines, especially C6:0, C8:0, C8:1, C12:1, C14:1, C16:0, C16:1, C18:1, and C20:4, were found to be elevated in CAD patients. This accumulation could be explained by a dysregulation in carnitine shuttle enzymes and by an inefficient beta-oxidation as previously demonstrated (34, 38, 40, 44, 56, 87–90). The main carnitine shuttle enzymes are carnitine palmitoyltransferase 1 (CPT1) and carnitine palmitoyltransferase 2 (CPT2), which are responsible for the conversion of acyl-CoA and carnitine to free CoA and acylcarnitine and the opposite reaction, respectively. The conversion of acyl-CoA to acylcarnitine allow fatty acids longer than 10 carbon atoms to be transported across the mitochondrial membrane for subsequent beta oxidation. In ischemic conditions, CPT1 activity is increased and CPT2 activity decreased, leading to an accumulation of medium- and long-chain acylcarnitines (Figure 5) (87). Furthermore, ischemia leads to an altered beta-oxidation, which may be attributed to impaired function of fatty acid oxidation enzymes or increased fatty acid oxidation relative to tricarboxylic acid (TCA) flux (12, 38), both leading to accumulation of acyl-CoA (12, 34, 47, 88, 91, 92). Excess acyl-CoA can be retroconverted to acylcarnitine, which can then be excreted via blood and urine, thus detoxifying mitochondria of excess carbons (Figure 5) (37, 39, 88, 93).

Our results are in line with previous findings. Within medium-chain acylcarnitines, hexanoylcarnitine (C6:0) was reported to be able to discriminate patients with cardiovascular diseases from clinically healthy controls (16). Likewise, octanoylcarnitine (C8:0) was associated with cardiovascular mortality and reduced heart function (17, 94). Within long-chain acylcarnitines, palmitoylcarnitine (C16:0) has been associated with heart failure (94), cardiovascular mortality in patients with stable angina pectoris (17) and cardiovascular events in very old individuals with previous history of CAD (15). Similarly, oleoylcarnitine (C18:1) was shown to be able to predict cardiovascular events in elderly individuals with previous history of CAD (15).

### The Interconnection of Short-Chain Acylcarnitine and BCAA Metabolism

We found elevated levels of several C3- and C5-acylcarnitine species, as well as of their precursors valine and isoleucine, in patients with CAD. Our results are consistent with those of previous studies, which found that elevated levels of circulating BCAAs (19, 28, 29) and short-chain acylcarnitines (19, 28) were associated with CAD and stroke in a population at high cardiovascular risk (95). BCAAs and acylcarnitines seem to interplay at different levels. First, chronic cardiac ischemia can disrupt the BCAA catabolism, leading to increased BCAA catabolism derivatives such as C3- and C5-acylcarnitines (38, 96, 97). Excess BCAAs can then impair fatty acid oxidation, which results in the accumulation of incompletely oxidized lipid species and acylcarnitines (5, 98).

Acetylcarnitine (C2:0), which is the most abundant circulating acylcarnitine (99), plays a central role in detoxifying mitochondria from excessive acetyl-CoA, the universal degradation product of all metabolic substrates (Figure 5) (93). Interestingly, we found that acetylcarnitine (C2:0) was not significantly associated with CAD phenotype and does not accumulate in CAD patients. As the acetyl-CoA is the main substrate of the TCA cycle, this observation implies that the capacity of the TCA cycle is not necessarily exceeded as previously postulated (12, 38, 98). Therefore, the globally elevated levels of acylcarnitines are likely due to impaired fatty acid oxidation and carnitine shuttle enzymes, rather than to reduced TCA flux.

### Hydroxylated and Dicarboxylic Acylcarnitines

The short-chain hydroxylated acylcarnitine hydroxybutyrylcarnitine (C4:0-OH) was found to be elevated in CAD. Accumulation of plasma hydroxybutyrylcarnitine (C4:0-OH) is used for the diagnosis and screening of patients with an inherited defect in the short-chain hydroxyacyl-CoA dehydrogenase (SCHAD), an enzyme of the mitochondrial fatty acid oxidation (100). This finding further supports an impaired beta oxidation in CAD.

Suberoylcarnitine (C8:0-DC) was positively associated with CAD phenotype. This is in line with the findings of Shah et al., which showed that a signature composed of short- and medium-chain dicarboxylic acylcarnitines was predictive of cardiovascular events in individuals with CAD (19). In addition to an alteration in mitochondrial fatty acid oxidation, elevated dicarboxylic acylcarnitine levels in CAD could indicate increased fatty acid omega-oxidation (101). Dicarboxylic acylcarnitines are byproducts of medium-chain dicarboxylic acids. The latter are the final products of microsomal omega-oxidation and of the subsequent peroxisomal beta-oxidation (102). Both mitochondria and peroxisomes perform fatty acid beta-oxidation but with different aims. Short-, medium and long-chain fatty acids are predominantly oxidized in mitochondria, whereas peroxisomes oxidize specific carboxylic acids such as very long-chain fatty acids, branched-chain fatty acids, bile acids, and fatty dicarboxylic acids (DCAs) (103, 104). The carnitine shuttle is then used to transport the end-products (acetyl-CoA, propionyl-CoA, and medium-chain acyl-CoA) from the peroxisome to the mitochondria for complete oxidation via the TCA cycle (Figure 5) (37, 38, 105). While the mitochondrial beta-oxidation is essential for catabolism and energy production, peroxisomal beta-oxidation is mainly involved in biosynthesis pathways (106). Altogether, in addition to impaired mitochondrial beta-oxidation, CAD patients also seem to have an altered peroxisomal and microsomal fatty acid oxidation (105).

### Acylcarnitines: Angels or Demons?

An accumulation of medium- and long-chain acylcarnitines can impair several regulatory mechanisms in mitochondria. First, fatty acids and glucose intermediates compete as metabolic substrates for energy production (Randle cycle) (42). An intramitochondrial accumulation of long-chain acylcarnitines inhibits pyruvate and lactate oxidation, leading to metabolic inflexibility (Figure 5) (42) or the incapacity to switch between substrate for energy production depending on their availability (41). Secondly, an excess of long-chain acylcarnitines compromises membrane function, induces electrophysiological alterations through modulation of calcium and potassium channels (contributing to cardiac arrhythmias), promotes insulin resistance and inflammation, inhibits oxidative phosphorylation and stimulates the production of reactive oxygen species (34, 37, 87, 88, 107).

A dysregulation in the BCAA catabolism, with a back-up of BCAAs and their byproducts, also has important consequences. The BCAA catabolism intermediates branched-chain keto acids (BCKAs) can be cytotoxic at high levels, promoting mitochondrial dysfunction, superoxide accumulation, and cardiomyocyte death, eventually leading to heart failure (96). Furthermore, an accumulation of BCAAs sensitizes the heart to ischemic injury (108) and contributes to cardiac dysfunction and remodeling following myocardial ischemia (109, 110). This can be explained by various mechanisms including inhibition of glucose metabolism (108) and activation of the mammalian target of rapamycin (mTOR) (109, 110). Altogether, BCAA catabolism seems to be disrupted in individuals with CAD, provoking an accumulation of BCAAs and their byproducts, favorizing mitochondrial dysfunction.

While an accumulation of BCAAs, medium- and long-chain acylcarnitines have deleterious consequences, research has indicated that some short-chain acylcarnitines could also have positive effects (37). Evidence has suggested that propionylcarnitine (C3) increases cellular carnitine content, stimulates pyruvate dehydrogenase activity and increases TCA cycle efficiency under hypoxia (111, 112). Therefore, supplementation in propionylcarnitine (C3) could be beneficial in the treatment of cardiovascular disorders (112). For L-carnitine, several studies have also reported a protective role on the myocardium by exerting anti-apoptotic effects in cardiomyocytes (94, 113). A protective role of L-carnitine within the myocardium is further supported by studies in individuals with genetic carnitine deficiency developing cardiomyopathies (114). Importantly, in our study, propionylcarnitine and L-carnitine were found to be elevated in CAD patients. Considering the potential beneficial effects of these carnitine species, elevated levels of circulating L-carnitine and proprionylcarnitine observed in CAD patients in our study could suggest an attempt of the body to adapt to chronic ischemia. This hypothesis remains to be further investigated.

### Can Acylcarnitines Replace Cholesterol in Clinical Practice?

Mitochondrial dysfunction is a major determinant of metabolic disease such as metabolic syndrome, non-alcoholic fatty liver disease and type 2 diabetes mellitus, conditions which are highly linked to increased risk of cardiovascular disease and myocardial infarction (115, 116). This supports the use of acyclarnitines as markers of mitochondrial function and cardio-metabolic risk. Additionally, understanding the acylcarnitine metabolism in CAD could lead to new treatment targets. Most research on the effects of ischemia on mitochondrial enzymes has been done in acute ischemic situations. This study showed that altered mitochondrial metabolism reflected in high levels of circulating carnitine, acylcarnitines and BCAAs is also a hallmark of CAD, a chronic ischemic situation. These findings should be further investigated at an enzymatic level and in model organisms. There has been emerging evidence that manipulating fuel supply and substrate consumption of the myocardium could have an impact on the development and progression of heart failure (117, 118), which often occurs in CAD. Therapeutic interventions with “metabolic” antianginal agents that suppress fatty acid oxidation and increase the oxidation of pyruvate in the mitochondria could reduce the ischemia-induced accumulation of long-chain fatty acid intermediates and other disruption in cardiac metabolism (119). Hypoxia, anoxia and ischemia might have different effects on the cardiac metabolism and these conditions should be clearly distinguished in future works (33).

### Strengths and Limitations

While previous studies analyzed L-carnitine and/or a limited number of acylcarnitines in patients with cardiometabolic diseases, we examined a large panel of acylcarnitine species and related BCAAs. This resulted in a thorough phenotyping of patients with CAD at the molecular species level (35 metabolites) comparing them to clinically healthy controls, which was rarely the case in previous research. Additionally, we conducted a detailed analysis of possible confounders and adjusted the regressions for those variables. Given the exploratory nature of this study, several limitations should be taken into consideration. First, as multiple organs usually contribute to the circulating pool of metabolites, it is difficult to identify the cellular origin, destination or subcellular localization of these metabolites (120). Therefore, the findings of the present study should be interpreted with caution when it comes to mechanistic explanations. Fortunately, it was recently shown that circulating acylcarnitine levels reflect cardiac tissue content of acylcarnitines (121), whereas elevated plasma levels of BCAAs reflect impaired BCAA catabolism in cardiac cells (122). These two facts support the rationale of the present study. Secondly, the cross-sectional nature of this study only allows for the establishment of associations, and not causality, between metabolites and CAD (123). While it is currently unknown if elevated level of circulating acylcarnitines is a consequence or a cause of ischemia-related cardiac damages, this question is of crucial relevance for the therapy of CAD and should be further investigated. Thirdly, the extent to which the reported associations represent the acylcarnitine and BCAA signature of CAD in females is unclear, as most enrolled patients were male (90.7%). This study likely did not capture the sex-specific metabolic signature of CAD. Fourthly, although serum samples were collected in a fasting state and regression analyses were adjusted for fasting time, the fasting duration might have been too short (6.7 h ± 3.0 h for healthy controls and 8.5 h ± 5.3 h for CAD patients). However, while circulating acylcarnitine levels have been shown to decrease up to 3 h after food intake (124) and increase after 12 h of fasting (125–127), the effect of a fasting time comprised between 3 and 12 h is unknown to the authors' best knowledge. Finally, we did not control for the amount and type of food intake, which is known to influence the metabolome (67).

## Conclusion

This study found elevated levels of circulating acylcarnitine and BCAA species in patients with CAD compared to clinically healthy individuals. Acylcarnitine species of all chain-lengths showed positive associations with CAD phenotype. Interestingly, associations between acylcarnitine species and CAD became stronger as the number of affected coronary arteries increased. Thus, circulating acylcarnitine levels might reflect CAD severity and should be considered as potential candidates to improve patients' stratification. Altogether, CAD is characterized, at a molecular species level, by elevated acylcarnitine and BCAA levels, thus implying impaired mitochondrial metabolism in cardiac cells.

## Data Availability Statement

All data presented in this study are available within the article and Supplementary Material.

## Ethics Statement

The studies involving human participants were reviewed and approved by Ethics Committee of North-Western and Central Switzerland. The patients/participants provided their written informed consent to participate in this study.

## Author Contributions

JG, JC, HG-A, DI, JW, RK, AS-T, and JI contributed to conception and design of the study. JC, JW, CK, GN, and RK collected the data. HG-A, RB, TT, and JI analyzed the serum samples for the metabolites. JG, JC, and DI performed the statistical analysis. JG and JC wrote the first draft of the manuscript. TT wrote section Biochemical Analysis of the manuscript. HG-A, DI, FC, LS, JW, CK, RK, HH, AS-T, and JI contributed to manuscript revision. JC, JI, and AS-T supervised the study. AS-T was responsible for the funding acquisition. All authors read and approved the submitted version.

## Funding

This study was funded by the Swiss National Science Foundation (grant nos. 182815 to AS-T and 316030_183377 to JI). This work was also supported by funds from Faculty of Biology and Medicine (FBM), University of Lausanne (UNIL).

## Conflict of Interest

The authors declare that the research was conducted in the absence of any commercial or financial relationships that could be construed as a potential conflict of interest.

## Publisher's Note

All claims expressed in this article are solely those of the authors and do not necessarily represent those of their affiliated organizations, or those of the publisher, the editors and the reviewers. Any product that may be evaluated in this article, or claim that may be made by its manufacturer, is not guaranteed or endorsed by the publisher.
