# Supplementary material for: Metabolic Impairment in Coronary Artery Disease: Elevated Serum Acylcarnitines Under the Spotlights
Source: Front Cardiovasc Med. 2021 Dec 16;8:792350. doi: 10.3389/fcvm.2021.792350 (PMC8716394; doi:10.3389/fcvm.2021.792350)
Supplement: Supplementary file 3 [file Data_Sheet_1.docx]

# *Supplementary Materials*

# Supplement statistical method – adjustments for regression

According to Tokarz and Adamski (1), the most phenotypically penetrant confounders affecting the metabolome are genetics, ethnicity, sex, age, nutrition, body mass index (BMI), physical activity, alcohol, smoking, stress, circadian rhythm, hormonal status, medication, lifestyle and disease. The traditional methods of adjusting for "potential confounders" may introduce additional associations and bias rather than minimizing them. This is why we used a directed acyclic graph (DAG) (2) based on the existing literature to identify the independent variables of the regression. Adjustments were then made for these possible confounding variables (see supplementary Figures 1 and 2). The online tool DAGitty V3.0 (3, 4) was used to create the DAG. For acylcarnitines, the regression was adjusted for age, sex, percentage of body fat (PBF), glycated hemoglobin (HbA1c), smoking habits, drugs (beta-blockers, calcium antagonists, angiotensin-converting-enzyme (ACE)-inhibitors and statins) as well as for the fasting and sampling time. Indeed studies have showed an increase in plasma acylcarnitines with age (5, 6), male sex (7), high PBF (8–10), diabetes mellitus (10–14) and active smoking (even though most of the effects can be reversed by smoking cessation) (15). Some medications are known to affect fatty acid metabolism and acylcarnitine release such as 1) lipid-lowering agents, e.g., statin and fibrates, which increase carnitine acetyltransferase activity (16, 17); or 2) β-blockers that decrease carnitine palmitoyltransferase 1 (CPT-1) activity and decrease organic cation/carnitine transporter 2 (OCTN2) expression (18, 19); or other anti-hypertensive medication such as amlodipine and losartan, which have been associated with decreased circulating levels of acylcarnitines (20). All the above-mentioned possible parameters are risk factors for coronary artery disease (CAD), except medications which treat this disease. Both risks factors and medication are associated with the exposure (CAD) and outcome (acylcarnitines). Hypertension is a risk factor for the development of CAD and was shown to be associated with higher plasma acylcarnitine levels (21). Because of the association with both the exposure (CAD) and outcome (acylcarnitine) in our DAG, the regression could potentially be adjusted for hypertension. We decided not to make this adjustment for the following reasons: 1) a well-treated hypertension cannot be taken into account the same way as an untreated hypertension 2) hypertension is very closely linked to atherosclerosis, and therefore to CAD 3) and we do not know if high acylcarnitine levels are the cause or the consequence of hypertension. We were not able to adjust for diet because the dietary habits of the participants were not known/controlled.

For branched-chain amino acids (BCAAs), the regression was adjusted for age, sex, muscle mass, smoking status as well as for the fasting and sampling time. Circulating amino acid profile differ with age and sex (1, 22–24) as well as with muscle mass (25, 26) and smoking status (27, 28). The diet of the participants was not controlled and could not be adjusted for.

Fasting and sampling time do not have an influence on CAD but they can be the cause of major variations in circulating metabolite levels (1, 29, 30). Therefore, we adjusted the regressions for fasting and sampling time.

# Supplementary Figures Caption


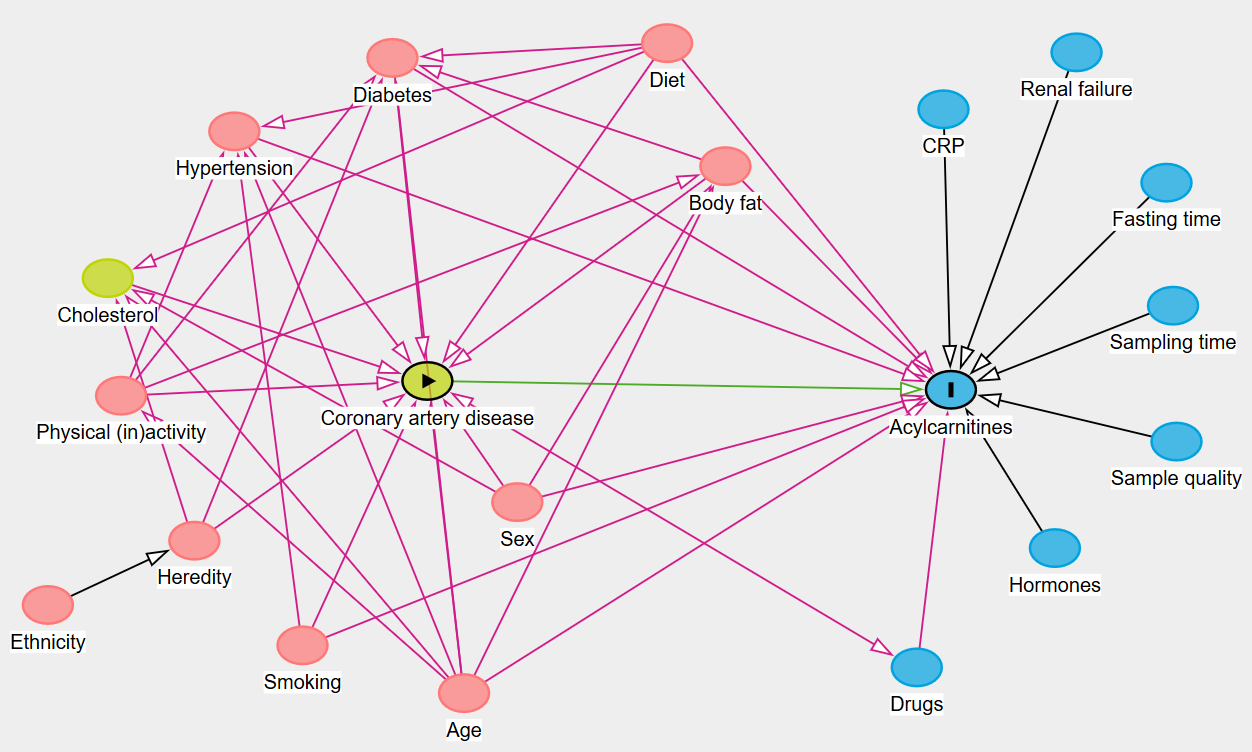


***Figure 1 Suppl.*** ***Directed acyclic graph (DAG) created with DAGitty v3.0 showing the relationships between acylcarnitines and coronary artery disease (CAD).*** *CAD was defined as the exposure and acylcarnitines as the outcome. The green circles are the ancestors of exposure, blue circles the ancestors of outcome and pink circles the ancestors of both exposure and outcome. The green line is the causal path and the pink lines are the biasing paths. DAGitty proposed age, body fat, diabetes, diet, drugs, hypertension, sex and smoking as minimal sufficient adjustment sets for estimating the total effect of coronary artery disease on acylcarnitines.*


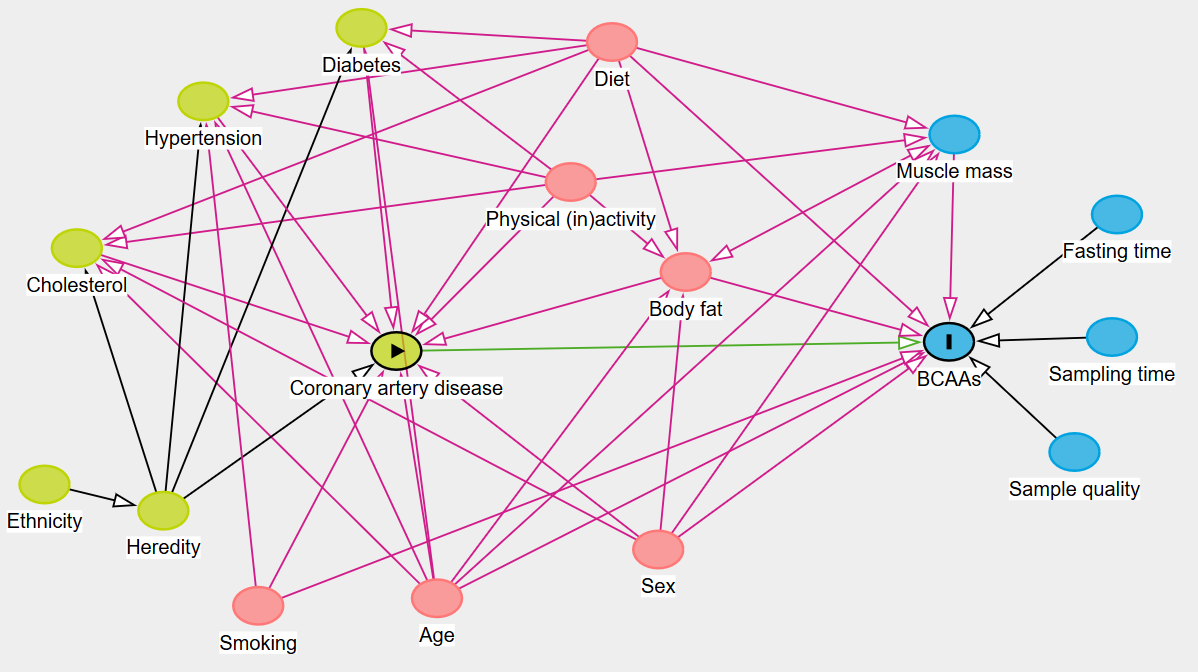


***Figure 2 Suppl.*** ***DAG created with DAGitty v3.0 showing the relationship between branched-chain amino acids (BCAAs) and coronary artery disease (CAD).*** *CAD was defined as the exposure and BCAAs as the outcome. The green circles are the ancestors of exposure, blue circles the ancestors of outcome and pink circles the ancestors of both exposure and outcome. The green line is the causal path and the pink lines are the biasing paths. DAGitty proposed age, body fat, diet, muscle mass, sex and smoking as minimal sufficient adjustment sets for estimating the total effect of coronary artery disease on BCAAs.*

*
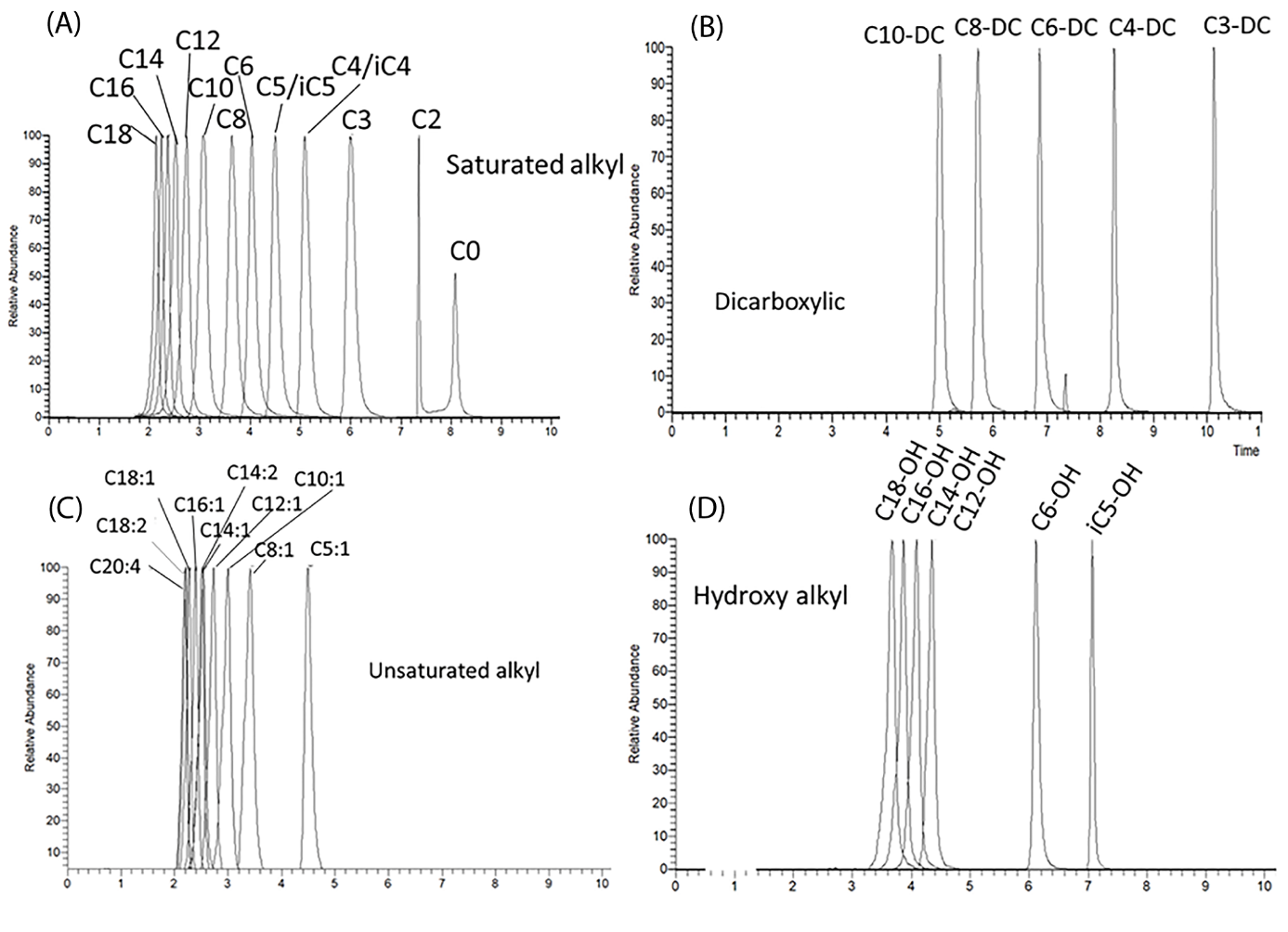
*

*Figure 3 Suppl. HILIC chromatographic separation of A) saturated acylcarnitines, B) dicarboxylic acylcarnitines, C) unsaturated acylcarnitines and D) hydroxylated acylcarnitines that were quantified in an absolute manner.*

# Supplementary Tables

***Table 1 Suppl.*** *Targeted metabolites. Abbreviations: C = number of carbon atoms of the acyl-group, DC = dicarboxyl, OH = Hydroxy, BCAA = branched-chain amino acid*

|  | **Abbre-viation** | **Targeted metabolites** | **Mass-to-Charge Ratio** | **Retention time (min)** | **Internal standards** |
| --- | --- | --- | --- | --- | --- |
|  | C0 | Carnitine | 162.11 | 8.02 | L-Carnitine-(N-methyl-d3), inner salt |
|  |  | Deoxycarnitine | 146.12 | 6.07 | γ-Butyrobetaine-d9 |
| Short-chain (n=10) | C2:0 | Acetylcarnitine | 204.12 | 5.98 | Acetyl-L-carnitine-(N-methyl-d3) |
|  | C3:0 | Propionylcarnitine | 218.14 | 5.08 | Propionyl-L-carnitine-(N-methyl-d3) |
|  | C3-DC (C4:0-OH) | Malonylcarnitine (hydroxybutyrylcarnitine) | 248.11 | 10.06 | Malonylcarnitine-d3 |
|  | C4:0 | Butyrylcarnitine | 232.15 | 4.48 | Butyryl-L-carnitine-(N-methyl-d3) |
|  | C4:0-OH | Hydroxybutyrylcarnitine | 248.15 | 7.52 | Hydroxybutyrylcarnitine-d3 |
|  | C4:1-O2 | O-succinylcarnitine | 262.13 | 8.23 | O-Succinyl-L-carnitine-d3 |
|  | C5:0 | Isovalerylcarnitine | 246.17 | 4.03 | Isovaleryl-L-carnitine-(N,N,N-trimethyl-d9) |
|  | C5:0-OH | 3-Hydroxyvalerylcarnitine | 262.16 | 6.90 | 3-Hydroxyisovaleryl-L-carnitine-(N-methyl-d3) |
|  | C5:1 | Tiglylcarnitine | 244.15 | 4.34 | Tiglylcarnitine-d3 |
|  | C5:1-O2 | Glutarylcarnitine | 276.14 | 7.71 | Glutaryl-L-carnitine-(N-methyl-d3) lithium salt |
| Medium-chain (n=13) | C6:0 | Hexanoylcarnitine | 260.19 | 3.66 | Hexanoyl-L-carnitine-(N-methyl-d3) |
|  | C6:1 | Hexenoylcarnitine | 258.17 | 3.86 | Hexanoyl-L-carnitine-(N-methyl-d3) |
|  | C6:0-OH | 3-Hydroxyhexanoylcarnitine | 276.18 | 5.96 | [(R)-3-Hydroxyhexadecanoyl]-L-carnitine-(methyl-d3) |
|  | C6:1-O2 | Adipoylcarnitine | 290.16 | 6.85 | Adipoylcarnitine-d3 |
|  | C8:0 | Octanoylcarnitine | 288.22 | 3.09 | Octanoyl-L-carnitine-(N-methyl-d3) |
|  | C8:0-DC | Suberoylcarnitine | 318.19 | 5.72 | Suberoylcarnitine-d3 |
|  | C8:1 | 2-Octenoylcarnitine | 286.20 | 3.28 | Octanoyl-L-carnitine-(N-methyl-d3) |
|  | C10:0 | Decanoylcarnitine | 316.25 | 2.76 | Decanoyl-L-carnitine-(N-methyl-d3) |
|  | C10:1 | Trans-2-decenoylcarnitine | 316.25 | 2.76 | Decanoyl-L-carnitine-(N-methyl-d3) |
|  | C10-DC | Sebacoylcarnitine | 346.22 | 5.01 | Sebacoyl-L-carnitine-d3 |
|  | C12:0 | Lauroylcarnitine (dodecanoylcarnitine) | 344.28 | 2.54 | Lauroyl-L-carnitine-(N,N,N-trimethyl-d9) |
|  | C12:0-OH | 3-Hydroxydodecanoylcarnitine | 360.27 | 4.25 | Lauroyl-L-carnitine-(N,N,N-trimethyl-d9) |
|  | C12:1 | Trans-2-dodecenoylcarnitne | 342.26 | 2.65 | Lauroyl-L-carnitine-(N,N,N-trimethyl-d9) |
| Long-chain (n=13) | C14:0 | Myristoylcarnitne (tetradecanoylcarnitine) | 372.31 | 2.37 | Myristoyl-L-carnitine-(N,N,N-trimethyl-d9) |
|  | C14:0-OH | 3-Hydroxytetradecanoylcarnitine | 388.31 | 3.99 | [(R)-3-Hydroxyhexadecanoyl]-L-carnitine-(methyl-d3) |
|  | C14:1 | Trans-2-tetradecenoylcarnitine | 370.30 | 2.48 | Myristoyl-L-carnitine-(N,N,N-trimethyl-d9) |
|  | C14:2 | Cis,cis-5,8-tetradecanedienoylcarnitine | 368.28 | 2.45 | Myristoyl-L-carnitine-(N,N,N-trimethyl-d9) |
|  | C16:0 | Palmitoylcarnitine (hexadecanoylcarnitine) | 400.34 | 2.25 | Palmitoyl-L-carnitine-(N-methyl-d3) |
|  | C16:0-OH | 3-Hydroxyhexadecanoylcarnitine | 416.34 | 3.77 | [(R)-3-Hydroxyhexadecanoyl]-L-carnitine-(methyl-d3) |
|  | C16:1 | Trans-2-hexadecenoylcarnitine | 398.33 | 2.34 | Palmitoyl-L-carnitine-(N-methyl-d3) |
|  | C17:0 | Heptadecanoylcarnitine | 414.36 | 2.19 | Palmitoyl-L-carnitine-(N-methyl-d3) |
|  | C18:0 | Stearoylcarnitine (octadecanoylcarnitine) | 428.37 | 2.14 | Stearoyl-L-carnitine-(N-methyl-d3) |
|  | C18:0-OH | 3-Hydroxyoctadecanoylcarnitine | 444.37 | 3.58 | [(R)-3-Hydroxyhexadecanoyl]-L-carnitine-(methyl-d3) |
|  | C18:1 | Oleoylcarnitine (octadecenoylcarnitine) | 426.36 | 2.17 | Oleoyl-L-carnitine-d3 Inner Salt |
|  | C18:2 | Cis,cis-9,12- octadecadienoylcarnitine | 424.34 | 2.22 | Stearoyl-L-carnitine-(N-methyl-d3) |
|  | C20:4 | Arachidonylcarnitine | 448.34 | 2.15 | Stearoyl-L-carnitine-(N-methyl-d3) |
| BCAA |  | Leucine | 132.10 | 7.21 | Leucine (13C6, 99%; 15N, 99%) |
|  |  | Isoleucine | 132.10 | 7.52 | Isoleucine (13C6, 99%; 15N, 99%) |
|  |  | Valine | 118.09 | 8.28 | Valine (13C5, 99%; 15N, 99%) |

***Table 2 Suppl****. β-coefficients and BH p-values of the associations between carnitine, acylcarnitines, branched-chain amino acids (BCAAs) and coronary artery disease (CAD).*

|  |  |  | **CAD** | | | **One vessel CAD** | | | **Two vessel CAD** | | | **Three vessel CAD** | | |
| --- | --- | --- | --- | --- | --- | --- | --- | --- | --- | --- | --- | --- | --- | --- |
|  | **Metabolites** | | **β-coefficient** | **95%CI** | **BH p-value** | **β-coefficient** | **95%CI** | **BH p-value** | **β-coefficient** | **95%CI** | **BH p-value** | **β-coefficient** | **95%CI** | **BH p-value** |
|  | C0 | Carnitine | 0.78 | 0.32-1.24 | 0.007 | 0.65 | 0.09-1.21 | 0.081 | 0.73 | 0.10-1.35 | 0.081 | 0.64 | 0.09-1.18 | 0.081 |
|  |  | Deoxycarnitine | 0.49 | -0.09-1.06 | 0.222 | 0.49 | -0.24-1.22 | 0.336 | 0.40 | -0.40-1.21 | 0.489 | 0.46 | -0.24-1.17 | 0.351 |
| Short-chain (n=10) | C2:0 | Acetylcarnitine | 0.65 | 0.07-1.24 | 0.086 | 0.25 | -0.46-0.96 | 0.634 | 0.40 | -0.39-1.19 | 0.482 | 0.90 | 0.21-1.59 | 0.046 |
|  | C3:0 | Propionylcarnitine | 0.74 | 0.26-1.22 | 0.016 | 0.58 | -0.01-1.16 | 0.139 | 0.52 | -0.14-1.17 | 0.244 | 0.78 | 0.20-1.35 | 0.038 |
|  | C3-DC (C4:0-OH) | Malonylcarnitine (Hydroxybutyryl-carnitine) | N/A | N/A | N/A | N/A | N/A | N/A | N/A | N/A | N/A | N/A | N/A | N/A |
|  | C4:0 | Butyrylcarnitine | 0.67 | 0.17-1.16 | 0.036 | 0.53 | -0.07-1.13 | 0.195 | 0.45 | -0.22-1.12 | 0.336 | 0.68 | 0.09-1.27 | 0.082 |
|  | C4:0-OH | Hydroxybutyryl-carnitine | 0.79 | 0.23-1.35 | 0.029 | 0.35 | -0.34-1.05 | 0.482 | 0.78 | 0.01-1.55 | 0.127 | 1.06 | 0.39-1.74 | 0.013 |
|  | C4:1-O2 | Succinylcarnitine | 0.55 | 0.00-1.10 | 0.136 | 0.42 | -0.25-1.09 | 0.375 | 0.29 | -0.46-1.03 | 0.606 | 0.67 | 0.01-1.32 | 0.124 |
|  | C5:0 | Isovalerylcarnitine | 0.69 | 0.20-1.18 | 0.029 | 0.46 | -0.15-1.06 | 0.268 | 0.74 | 0.07-1.41 | 0.094 | 0.65 | 0.07-1.24 | 0.093 |
|  | C5:0-OH | Hydroxyvaleryl-carnitine | 0.75 | 0.22-1.27 | 0.027 | 0.63 | -0.01-1.28 | 0.139 | 0.63 | -0.08-1.35 | 0.193 | 0.68 | 0.05-1.31 | 0.103 |
|  | C5:1 | Tiglylcarnitine | 0.93 | 0.43-1.42 | 0.003 | 0.85 | 0.24-1.47 | 0.033 | 0.80 | 0.11-1.48 | 0.081 | 0.95 | 0.35-1.55 | 0.013 |
|  | C5:1-O2 | Glutarylcarnitine | 0.69 | 0.17-1.21 | 0.039 | 0.58 | -0.05-1.21 | 0.170 | 0.53 | -0.18-1.23 | 0.273 | 0.73 | 0.12-1.35 | 0.076 |
| Medium-chain (n=13) | C6:0 | Hexanoylcarnitine | 1.02 | 0.46-1.58 | 0.004 | 0.66 | -0.04-1.35 | 0.155 | 0.76 | -0.01-1.53 | 0.138 | 1.26 | 0.59-1.94 | 0.003 |
|  | C6:1 | Hexenoylcarnitine | N/A | N/A | N/A | N/A | N/A | N/A | N/A | N/A | N/A | N/A | N/A | N/A |
|  | C6:0-OH | Hydroxyhexanoyl-carnitine | 0.34 | -0.19-0.87 | 0.386 | 0.04 | -0.61-0.70 | 0.932 | 0.25 | -0.48-0.98 | 0.638 | 0.56 | -0.08-1.20 | 0.201 |
|  | C6:1-O2 | Adipoylcarnitine | 0.65 | 0.10-1.21 | 0.069 | 0.47 | -0.20-1.14 | 0.316 | 0.28 | -0.46-1.03 | 0.606 | 0.88 | 0.22-1.53 | 0.040 |
|  | C8:0 | Octanoylcarnitine | 0.86 | 0.30-1.42 | 0.016 | 0.37 | -0.32-1.05 | 0.471 | 0.75 | -0.01-1.52 | 0.138 | 1.27 | 0.60-1.94 | 0.003 |
|  | C8:0-DC | Suberoylcarnitine | 0.75 | 0.19-1.32 | 0.039 | 0.39 | -0.31-1.09 | 0.439 | 0.64 | -0.14-1.42 | 0.230 | 0.95 | 0.26-1.63 | 0.033 |
|  | C8:1 | Octenoylcarnitine | 0.87 | 0.32-1.43 | 0.013 | 0.80 | 0.12-1.49 | 0.081 | 0.61 | -0.15-1.37 | 0.242 | 0.93 | 0.26-1.60 | 0.033 |
|  | C10:0 | Decanoylcarnitine | 0.79 | 0.24-1.34 | 0.025 | 0.22 | -0.46-0.89 | 0.663 | 0.77 | 0.02-1.52 | 0.121 | 1.28 | 0.62-1.94 | 0.002 |
|  | C10:1 | Decenoylcarnitine | 0.75 | 0.21-1.30 | 0.032 | 0.34 | -0.33-1.02 | 0.482 | 0.48 | -0.27-1.23 | 0.358 | 1.10 | 0.44-1.76 | 0.008 |
|  | C10-DC | Sebacoylcarnitine | N/A | N/A | N/A | N/A | N/A | N/A | N/A | N/A | N/A | N/A | N/A | N/A |
|  | C12:0 | Lauroylcarnitine (Dodecanoyl-carnitine) | 0.74 | 0.20-1.27 | 0.032 | -0.01 | -0.64-0.63 | 0.982 | 0.87 | 0.17-1.58 | 0.061 | 1.42 | 0.80-2.04 | 0.0003 |
|  | C12:0-OH | Hydroxy-dodecanoyl-carnitine | 0.66 | 0.13-1.19 | 0.052 | 0.28 | -0.38-0.94 | 0.557 | 0.61 | -0.12-1.34 | 0.223 | 1.08 | 0.44-1.72 | 0.007 |
|  | C12:1 | Dodecenoylcarnitne | 0.82 | 0.29-1.35 | 0.016 | 0.23 | -0.42-0.87 | 0.634 | 0.84 | 0.13-1.56 | 0.078 | 1.31 | 0.68-1.93 | 0.001 |
| Long-chain (n=13) | C14:0 | Myristoylcarnitine (Tetradecanoyl-carnitine) | 0.65 | 0.11-1.19 | 0.063 | 0.09 | -0.57-0.76 | 0.860 | 0.90 | 0.16-1.63 | 0.066 | 1.16 | 0.51-1.80 | 0.004 |
|  | C14:0-OH | Hydroxy-tetradecanoyl-carnitine | N/A | N/A | N/A | N/A | N/A | N/A | N/A | N/A | N/A | N/A | N/A | N/A |
|  | C14:1 | Tetradecenoyl-carnitine | 0.87 | 0.35-1.38 | 0.008 | 0.25 | -0.37-0.88 | 0.578 | 0.96 | 0.27-1.65 | 0.033 | 1.43 | 0.83-2.04 | 0.0003 |
|  | C14:2 | Tetradecanedienoylcarnitine | 0.60 | 0.07-1.12 | 0.081 | 0.06 | -0.58-0.70 | 0.910 | 0.57 | -0.15-1.28 | 0.243 | 1.14 | 0.51-1.76 | 0.004 |
|  | C16:0 | Palmitoylcarnitine (Hexadecanoyl-carnitine) | 1.02 | 0.51-1.52 | 0.002 | 0.64 | 0.01-1.28 | 0.124 | 1.30 | 0.60-2.00 | 0.003 | 1.16 | 0.54-1.78 | 0.003 |
|  | C16:0-OH | Hydroxy-hexadecanoyl-carnitine | N/A | N/A | N/A | N/A | N/A | N/A | N/A | N/A | N/A | N/A | N/A | N/A |
|  | C16:1 | Hexadecenoyl-carnitine | 0.96 | 0.46-1.47 | 0.003 | 0.48 | -0.14-1.10 | 0.257 | 1.12 | 0.43-1.81 | 0.011 | 1.35 | 0.74-1.95 | 0.0004 |
|  | C17:0 | Heptadecanoyl-carnitine | 0.61 | 0.04-1.17 | 0.104 | 0.34 | -0.38-1.05 | 0.519 | 0.67 | -0.13-1.46 | 0.222 | 0.77 | 0.07-1.46 | 0.097 |
|  | C18:0 | Stearoylcarnitine (Octadecanoyl-carnitine) | -0.51 | -1.04-0.01 | 0.148 | -0.95 | -1.60-  -0.29 | 0.025 | -0.49 | -1.22-0.24 | 0.335 | -0.22 | -0.86-0.42 | 0.638 |
|  | C18:0-OH | Hydroxy-octadecanoyl-carnitine | N/A | N/A | N/A | N/A | N/A | N/A | N/A | N/A | N/A | N/A | N/A | N/A |
|  | C18:1 | Oleoylcarnitine (Octadecenoyl-carnitine) | 0.89 | 0.38-1.41 | 0.006 | 0.52 | -0.12-1.17 | 0.238 | 1.14 | 0.42-1.86 | 0.013 | 1.11 | 0.49-1.74 | 0.005 |
|  | C18:2 | Octadecadienoyl-carnitine | -0.05 | -0.57-0.48 | 0.907 | -0.34 | -1.00-0.33 | 0.482 | 0.15 | -0.59-0.88 | 0.798 | 0.13 | -0.51-0.78 | 0.798 |
|  | C20:4 | Arachidonyl-carnitine | 0.81 | 0.32-1.30 | 0.009 | 0.70 | 0.08-1.32 | 0.088 | 0.85 | 0.16-1.54 | 0.060 | 0.91 | 0.31-1.51 | 0.018 |

|  |  | **CAD** | | | **One vessel CAD** | | | **Two vessel CAD** | | | **Three vessel CAD** | | |
| --- | --- | --- | --- | --- | --- | --- | --- | --- | --- | --- | --- | --- | --- |
| **Metabolites** | | **β-coefficient** | **95%CI** | **BH p-value** | **β-coefficient** | **95%CI** | **BH p-value** | **β-coefficient** | **95%CI** | **BH p-value** | **β-coefficient** | **95%CI** | **BH p-value** |
| BCAA | Leucine | 0.32 | -0.09-0.74 | 0.258 | 0.10 | -0.54-0.73 | 0.895 | 0.48 | -0.18-1.13 | 0.322 | 0.38 | -0.12-0.89 | 0.322 |
|  | Isoleucine | 0.52 | 0.12-0.93 | 0.046 | 0.30 | -0.30-0.90 | 0.523 | 0.58 | -0.05-1.21 | 0.191 | 0.59 | 0.10-1.08 | 0.078 |
|  | Valine | 0.55 | 0.15-0.94 | 0.046 | 0.27 | -0.32-0.73 | 0.542 | 0.73 | 0.12-1.35 | 0.078 | 0.67 | 0.19-1.15 | 0.060 |

# References

1. Tokarz J, Adamski J. “Confounders in metabolomics,”. In: *Metabolomics for Biomedical Research*. Elsevier (2020). p. 17–32.

2. Shrier I, Platt RW. Reducing bias through directed acyclic graphs. *BMC Med Res Methodol* (2008) **8**:70. doi:10.1186/1471-2288-8-70

3. Textor J, van der Zander B, Gilthorpe MS, Liskiewicz M, Ellison GT. Robust causal inference using directed acyclic graphs: the R package 'dagitty'. *Int. J. Epidemiol.* (2016) **45**:1887–94. doi:10.1093/ije/dyw341

4. *DAGitty - draw and analyze causal diagrams* [cited 2021 Sep 07]. Available from: http://www.dagitty.net/

5. Huynh K, Barlow CK, Jayawardana KS, Weir JM, Mellett NA, Cinel M, et al. High-throughput plasma lipidomics: Detailed mapping of the associations with cardiometabolic risk factors. *Cell Chem Biol* (2019) **26**:71-84. doi:10.1016/j.chembiol.2018.10.008

6. Zachery R. Jarrell, M. Ryan Smith, Xin Hu, Michael Orr, Ken H. Liu, Arshed A. Quyyumi, et al. Plasma acylcarnitine levels increase with healthy aging. *Aging-US* (2020) **12**:13555–70. doi:10.18632/aging.103462

7. Mittelstrass K, Ried JS, Yu Z, Krumsiek J, Gieger C, Prehn C, et al. Discovery of sexual dimorphisms in metabolic and genetic biomarkers. *PLoS Genet* (2011) **7**:e1002215. doi:10.1371/journal.pgen.1002215

8. Baek SH, Kim M, Kim M, Kang M, Yoo HJ, Lee NH, et al. Metabolites distinguishing visceral fat obesity and atherogenic traits in individuals with overweight. *Obesity (Silver Spring)* (2017) **25**:323–31. doi:10.1002/oby.21724

9. Boulet MM, Chevrier G, Grenier-Larouche T, Pelletier M, Nadeau M, Scarpa J, et al. Alterations of plasma metabolite profiles related to adipose tissue distribution and cardiometabolic risk. *Am J Physiol Endocrinol Metab* (2015) **309**:E736-46. doi:10.1152/ajpendo.00231.2015

10. Mai M, Tönjes A, Kovacs P, Stumvoll M, Fiedler GM, Leichtle AB. Serum levels of acylcarnitines are altered in prediabetic conditions. *PLoS One* (2013) **8**:e82459. doi:10.1371/journal.pone.0082459

11. Adams SH, Hoppel CL, Lok KH, Zhao L, Wong SW, Minkler PE, et al. Plasma acylcarnitine profiles suggest incomplete long-chain fatty acid beta-oxidation and altered tricarboxylic acid cycle activity in type 2 diabetic African-American women. *J Nutr* (2009) **139**:1073–81. doi:10.3945/jn.108.103754

12. Bouchouirab F-Z, Fortin M, Noll C, Dubé J, Carpentier AC. Plasma palmitoyl-carnitine (AC16:0) is a marker of increased postprandial nonesterified incomplete fatty acid oxidation rate in adults with type 2 diabetes. *Can J Diabetes* (2018) **42**:382-388.e1. doi:10.1016/j.jcjd.2017.09.002

13. Koves TR, Ussher JR, Noland RC, Slentz D, Mosedale M, Ilkayeva O, et al. Mitochondrial overload and incomplete fatty acid oxidation contribute to skeletal muscle insulin resistance. *Cell Metab* (2008) **7**:45–56. doi:10.1016/j.cmet.2007.10.013

14. Mihalik SJ, Goodpaster BH, Kelley DE, Chace DH, Vockley J, Toledo FG, et al. Increased levels of plasma acylcarnitines in obesity and type 2 diabetes and identification of a marker of glucolipotoxicity. *Obesity (Silver Spring)* (2010) **18**:1695–700. doi:10.1038/oby.2009.510

15. Lacruz ME, Kluttig A, Tiller D, Medenwald D, Giegling I, Rujescu D, Prehn C, Adamski J, Frantz S, Greiser KH, Emeny RT, Kastenmüller G, Haerting J. Cardiovascular risk factors associated with blood metabolite concentrations and their alterations during a 4-year period in a population-based cohort. *Circ Cardiovasc Genet.* (2016):487–94. doi:10.22334/jbhost.v6i2.217.s47

16. Bhuiyan J, Seccombe DW. The effects of 3-hydroxy-3-methylglutaryl-CoA reductase inhibition on tissue levels of carnitine and carnitine acyltransferase activity in the rabbit. *Lipids* (1996) **31**:867–70. doi:10.1007/BF02522982

17. Iacobazzi V, Convertini P, Infantino V, Scarcia P, Todisco S, Palmieri F. Statins, fibrates and retinoic acid upregulate mitochondrial acylcarnitine carrier gene expression. *Biochem Biophys Res Commun* (2009) **388**:643–7. doi:10.1016/j.bbrc.2009.08.008

18. Panchal AR, Stanley WC, Kerner J, Sabbah HN. Beta-receptor blockade decreases carnitine palmitoyl transferase I activity in dogs with heart failure. *Journal of cardiac failure* (1998) **4**:121–6. doi:10.1016/S1071-9164(98)90252-4

19. Sharma V, McNeill JH. Parallel effects of β-adrenoceptor blockade on cardiac function and fatty acid oxidation in the diabetic heart: Confronting the maze. *World J Cardiol* (2011) **3**:281–302. doi:10.4330/wjc.v3.i9.281

20. Hiltunen TP, Rimpelä JM, Mohney RP, Stirdivant SM, Kontula KK. Effects of four different antihypertensive drugs on plasma metabolomic profiles in patients with essential hypertension. *PLoS One* (2017) **12**:e0187729. doi:10.1371/journal.pone.0187729

21. Mels CM, Schutte AE, Erasmus E, Huisman HW, Schutte R, Fourie CM, et al. L-carnitine and long-chain acylcarnitines are positively correlated with ambulatory blood pressure in humans: the SABPA study. *Lipids* (2013) **48**:63–73. doi:10.1007/s11745-012-3732-8

22. Foroumandi E, Alizadeh M, Kheirouri S. Age-dependent changes in plasma amino acids contribute to alterations in glycoxidation products. *J Med Biochem* (2018) **37**:426–33. doi:10.1515/jomb-2017-0065

23. Guevara-Cruz M, Vargas-Morales JM, Méndez-García AL, López-Barradas AM, Granados-Portillo O, Ordaz-Nava G, et al. Amino acid profiles of young adults differ by sex, body mass index and insulin resistance. *Nutr Metab Cardiovasc Dis* (2018) **28**:393–401. doi:10.1016/j.numecd.2018.01.001

24. Rist MJ, Roth A, Frommherz L, Weinert CH, Krüger R, Merz B, et al. Metabolite patterns predicting sex and age in participants of the Karlsruhe Metabolomics and Nutrition (KarMeN) study. *PLoS One* (2017) **12**:e0183228. doi:10.1371/journal.pone.0183228

25. Jourdan C, Petersen A-K, Gieger C, Döring A, Illig T, Wang-Sattler R, et al. Body fat free mass is associated with the serum metabolite profile in a population-based study. *PLoS One* (2012) **7**:e40009. doi:10.1371/journal.pone.0040009

26. Murphy RA, Moore SC, Playdon M, Meirelles O, Newman AB, Milijkovic I, et al. Metabolites associated with lean mass and adiposity in older black men. *J Gerontol A Biol Sci Med Sci* (2017) **72**:1352–9. doi:10.1093/gerona/glw245

27. Lacruz ME, Kluttig A, Tiller D, Medenwald D, Giegling I, Rujescu D, et al. Cardiovascular risk factors associated with blood metabolite concentrations and their alterations during a 4-year period in a population-based cohort. *Circ Cardiovasc Genet* (2016) **9**:487–94. doi:10.1161/CIRCGENETICS.116.001444

28. Tomoda K, Yoshikawa M, Kubo K, Koyama N, Yamamoto Y, Kimura H. Effects of cigarettes smoke on branched chain amino acids (BCAA) levels in plasma and skeletal muscles in rats. *J Toxicol Sci.* (2014) **39**:331-7. doi:10.2131/jts.39.331

29. Ang JE, Revell V, Mann A, Mäntele S, Otway DT, Johnston JD, et al. Identification of human plasma metabolites exhibiting time-of-day variation using an untargeted liquid chromatography-mass spectrometry metabolomic approach. *Chronobiol Int* (2012) **29**:868–81. doi:10.3109/07420528.2012.699122

30. Feigin R.D., Beisel W.R., Wannemacher R.W. Rhythmicity of plasma amino acids and relation to dietary intake. *The American Journal of Clinical Nutrition* (1971) **24**:329–41. doi:10.1093/ajcn/24.3.329
